# Aggressive Diffuse Leptomeningeal Glioneuronal Tumor in a Pediatric Patient Presenting With Mismatch Repair Gene Mutations

**DOI:** 10.7759/cureus.47905

**Published:** 2023-10-29

**Authors:** Alexander Torres-Rey, Juan Vigo-Prieto, Orlando De Jesus

**Affiliations:** 1 Neurosurgery, University of Puerto Rico, Medical Sciences Campus, San Juan, PRI

**Keywords:** mutations, mismatch repair genes, hyperproteinorrachia, hydrocephalus, genetics, diffuse leptomeningeal glioneuronal tumor

## Abstract

Diffuse leptomeningeal glioneuronal tumor (DLGNT) is a rare primary central nervous system tumor. We present the case of a five-year-old male patient with a rapid progression of a thoracic DLGNT. Initial presentation and workup confirmed acute communicating hydrocephalus requiring a ventriculoperitoneal shunt. Cerebrospinal fluid analysis showed hyperproteinorrachia. Additional workup demonstrated an intramedullary mass at the conus medullaris associated with leptomeningeal enhancement. A T10-T12 laminoplasty with tumor resection was performed. Immunohistochemistry was positive for glial fibrillary acid protein and synaptophysin, with a negative epithelial membrane antigen. The tumor had a Ki67 proliferation index of 9%. Gene tumor analysis revealed the presence of the *KIAA1549*-*BRAF* gene fusion. The tumor expressed *MSH6*, *MLH1*, *MSH2*, and *PMS2* mismatch repair gene mutations. Multiple subsequent shunt revisions were performed due to malfunction secondary to the hyperproteinorrachia. Follow-up studies showed extensive brain and spinal nodular cystic lesions associated with extensive leptomeningeal spread of disease. The patient received chemotherapy but died due to disease progression.

This case report described a rapidly progressive and aggressive DLGNT in a pediatric patient presenting mismatch repair gene mutations. Due to hyperproteinorrachia, shunt revisions are frequently needed in these patients. Even though DLGNT pathology can depict a low-grade tissue, some tumors behave aggressively with minimal significant response to medical and surgical treatments. Mutations of mismatch repair genes *MSH6*, *MLH1*, *MSH2*, and *PMS2* may be associated with more aggressive tumors.

## Introduction

Diffuse leptomeningeal glioneuronal tumor (DLGNT) is a rare primary central nervous system tumor. Its descriptive term was first used by Gardiman et al. in 2010 [1]. The World Health Organization (WHO) classified it as a distinguished entity in 2016 and a few years later as a specific tumor [2-4]. A WHO grade has yet to be assigned. In most cases, the tumor behaves like a low-grade tumor with prolonged overall survival; however, several cases with aggressive clinical courses have been described [5]. Existing reports are insufficient to develop adequate management guidelines and establish prognostic factors [5].

DLGNT is usually diagnosed in children, although adult cases have also been reported [5]. The clinical presentation of DLGNT is often nonspecific, which may include symptoms of hydrocephalus, spinal cord compression, and seizures. Signs and symptoms related to hydrocephalus are the most common clinical onset presentation [5]. Communicating hydrocephalus is secondary to the high protein content of the cerebrospinal fluid (CSF), creating an absorption deficiency [6]. This case report described a rare progressive and aggressive case of DLGNT in a five-year-old patient with acute communicating hydrocephalus and hyperproteinorrachia presenting with mutations in the mismatch repair genes.

## Case presentation

A five-year-old male patient was brought by his mother to the Emergency Department for evaluation after he had been complaining for one month of intermittent frontal headaches, nausea, and emesis, mainly occurring in the mornings. Neurological examination showed upward gazed palsy, convergence retraction nystagmus, and pupillary hyporeflexia (Parinaud’s syndrome). A contrast-enhanced brain MRI showed moderate communicating ventriculomegaly without any associated lesion. A lumbar puncture was performed for CSF analysis, and a temporary lumbar drainage was inserted to improve the symptoms. The CSF analysis revealed a protein level of 163 mg/dL (reference: 15-45 mg/dL) and a glucose level of 34 mg/dL. The white blood cell count was normal, and the CSF culture was negative. CSF cytology reported a few cohesive groups of atypical cells showing hyperchromatic nuclei, granular chromatin, and nuclei crowding.

**Figure 1:**
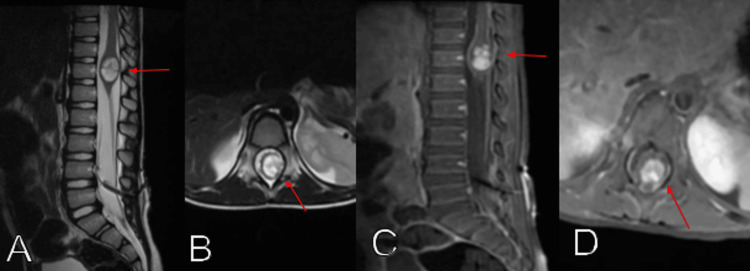
Spine MRI T2-weighted image, sagittal (A) and axial (B) views, and T1-weighted image with gadolinium, sagittal (C) and axial (D) views, showing an oval-shaped intramedullary mass (red arrows) at the T12 level causing expansion of the conus medullaris.

A spinal MRI, with and without contrast, showed a well-defined oval-shaped intramedullary mass at the T12 level, measuring 1.3 cm anteroposteriorly, 1.3 cm transversely, and 2.0 cm craniocaudally, displaying avid contrast enhancement (Figure 1). There was an expansion of the conus medullaris associated with interrupted leptomeningeal enhancement contour of the cervicothoracic cord and conus medullaris. A T10-T12 laminoplasty with tumor excision was performed. Immunohistochemistry was positive for glial fibrillary acid protein and synaptophysin, with a negative epithelial membrane antigen. The tumor had a Ki67 proliferation index of 9%. Gene tumor analysis revealed the presence of the *KIAA1549*-*BRAF* gene fusion. It was negative for *IDH1*, *IDH2*, and *NTRK1*/*2*/*3*. The tumor expressed *MSH6*, *MLH1*, *MSH2*, and *PMS2* mismatch repair gene mutations. The diagnosis of DLGNT was established based on the combination of the surgical tissue biopsy, the molecular and genetic profile, CSF sample results, and MRI scans showing evidence of the progression of the disease.

**Figure 2:**
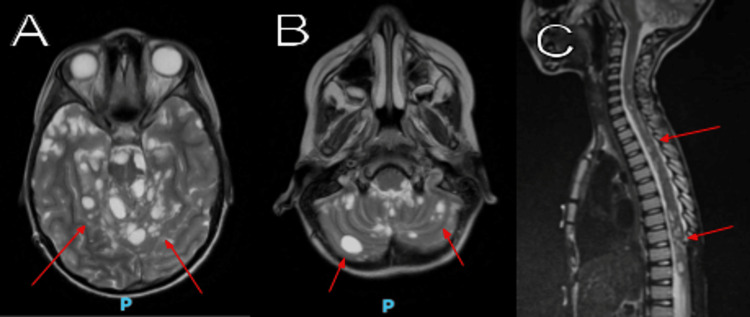
Brain and spine MRI (axial T2 propeller views) demonstrate numerous cystic tumoral lesions (red arrows) throughout the brain (A), cerebellum (B), and spine (C).

**Figure 3:**
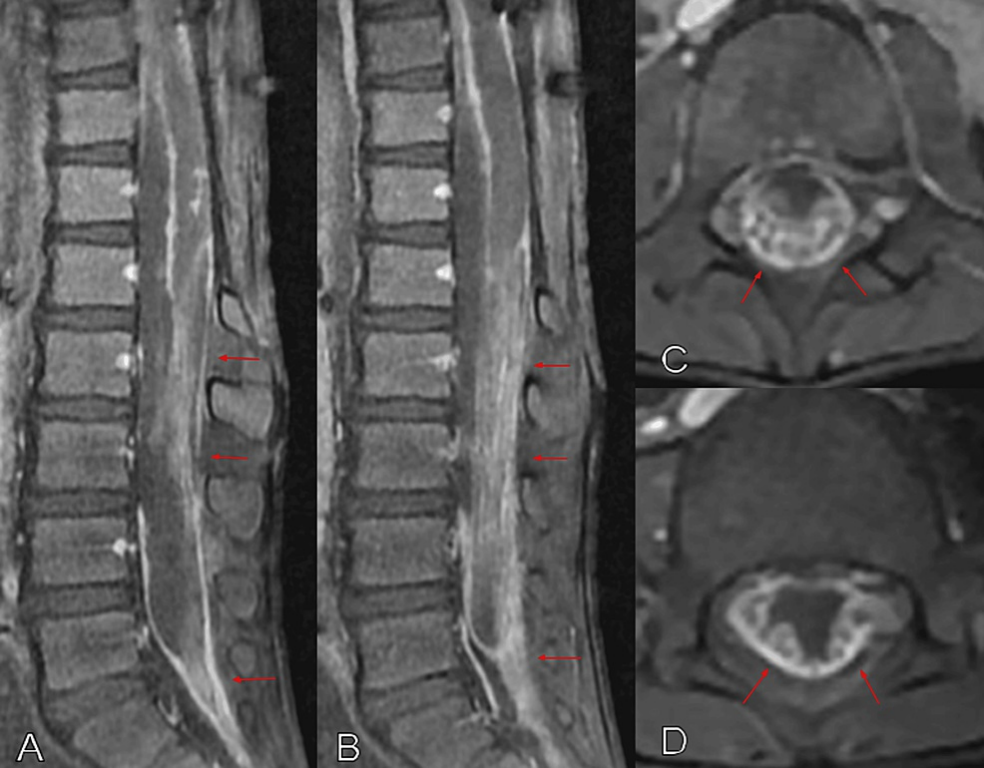
Lumbar MRI in sagittal (A, B) and axial (C, D) T1 views with gadolinium showing the extensive leptomeningeal spread of disease throughout the spine (red arrows).

A ventriculoperitoneal shunt was placed a few days later because of the communicating hydrocephalus. Multiple shunt revisions were performed due to valve malfunction caused by the markedly elevated CSF protein. Subsequent CSF samples showed elevated protein levels up to 537 mg/dL. The patient received chemotherapy protocol with vincristine and carboplatin, with the addition of vinblastine at the later stage of the disease. Follow-up brain and spine MRIs with and without contrast revealed numerous non-enhancing cystic lesions throughout the brain, brainstem, cerebellum, and spine (Figure 2). Spine MRI with and without contrast showed nodular meningeal thickening, associated with an extensive spinal leptomeningeal spread of disease (Figure 3). Unfortunately, the patient died 21 months after his initial presentation due to disease progression.

## Discussion

This report presented the case of a patient with a low-grade glial cell tumor at the conus medullaris associated with leptomeningeal enhancement. The patient manifested episodes of headaches, emesis, and hypoactivity secondary to hydrocephalus one month before the evaluation. Leptomeningeal dissemination through the CSF is unusual and occurs predominantly in medulloblastomas, ependymoblastomas, central neuroblastomas, ependymomas, germ cell tumors, and high-grade gliomas.

Children are most commonly affected by DLGNT, with the frequently being twice males [5,7]. Clinical presentation can be variable because of its extremely low incidence. Symptoms can vary depending on the rate at which the tumor is growing and the specific neural structures involved. Radiological imaging can demonstrate leptomeningeal enhancement, discrete intramedullary spinal masses, communicating hydrocephalus, or combinations of these findings [5]. DLGNT can be misdiagnosed for infectious or inflammatory pathologies [8]. Communicating hydrocephalus is the most frequently reported clinical finding and may be a direct result of leptomeningeal membrane thickening, altered CSF circulation, and impaired reabsorption [9]. Procedures for CSF diversion are frequently required in these patients to treat the hydrocephalus. In DLGNT, the CSF is associated with elevated protein levels, high cell counts, and CSF viscosity alterations. These factors contribute to an increase in the resistance to CSF flow and CSF sedimentation and subsequent valve malfunction.

These tumors contain recurrent genetic alterations, with *KIAA1549*-*BRAF* gene fusion being the most frequent [10,11]. A 1p deletion is frequently identified [5,10,11]. This case presents a rare mismatch repair gene mutation associated with Lynch syndrome, which has never been reported in patients with DLGNT. Lynch syndrome has been previously associated with an anaplastic glioneuronal tumor with a mutation in the mismatch repair genes *MSH2* and *MSH6* [12]. Some hypermutated gliomas have also been found to contain mutations in the mismatch repair genes [13]. Our patient’s tumor presented a mutation in four mismatch repair genes associated with Lynch syndrome.

Chemotherapy is associated with prolonged survival and should constitute the principal treatment regimen [5,14,15]. Radiotherapy’s role remains uncertain and should not be recommended as initial treatment considering insufficient data, the potential side effects, and the evidence of prolonged disease control in non-radiated patients [5]. Kinase inhibitors have been recently used with promising results [16]. Wiśniewski et al. showed that Ki-67 greater than 7% was the most important prognostic factor for overall survival in DLGNT [17]. Our case supports this observation, as the patient had a Ki67 proliferation index of 9% and died 21 months after his initial presentation due to disease progression. Jiang et al. showed that *KIAA1549*-*BRAF* positive patients had significantly higher overall survival than negative ones [18]. Although our patient had the *KIAA1549*-*BRAF* gene fusion, the high proliferation index and mutations in the mismatch repair protein genes may have contributed to the rapid disease progression.

## Conclusions

This case described an aggressive and progressive DLGNT associated with hyperproteinorrachia, which presented with acute communicating hydrocephalus. Due to hyperproteinorrachia, shunt revisions are frequently needed in patients with DLGNT; therefore, they should be monitored closely for evidence of shunt malfunction. Even though DLGNT pathology can depict a low-grade tissue, some tumors behave aggressively with minimal significant response to medical and surgical treatments. Mutations of mismatch repair genes *MSH6*, *MLH1*, *MSH2*, and *PMS2* may be associated with more aggressive tumors. More research for these tumors can delineate focused therapy and management, improving prognosis and outcomes.
